# *Notes from the Field:* Early-Season Human Plague Transmitted from an Infected Cat — Oregon, January 2024

**DOI:** 10.15585/mmwr.mm7426a2

**Published:** 2025-07-17

**Authors:** Emilio DeBess, Kelly Coyle, Richard Fawcett, Ali K. Hamade, Paul R. Cieslak

**Affiliations:** ^1^Oregon Health Authority, Portland, Oregon; ^2^Deschutes County Health Services, Bend, Oregon.

**Summary**

**What is known about this topic?**

Plague is caused by the bacterium *Yersinia pestis,* which is transmitted primarily through fleas from rodents. This case highlights an off-season transmission of plague. Plague is most often identified during May–August.

**What is added by this report?**

An Oregon man sought care at an emergency department for signs and symptoms of plague on January 30, 2024, the earliest calendar date of plague recorded in the state’s history, possibly indicating a shift in the seasonality of plague incidence. The patient did not have direct contact with rodents, but did have contact with his infected cat after cutting his finger.

**What are the implications for public health practice?**

Public health messaging and diagnostic efforts regarding plague are warranted year-round in areas with endemic disease.

Plague is caused by the bacterium *Yersinia pestis*. *Y. pestis* is transmitted primarily through the bite of an infected rodent flea or handling of infected animals. Plague is a rare but potentially life-threatening illness in the western United States, occurring in bubonic, septicemic, or pneumonic forms, primarily affecting rural populations, and is treatable with antibiotics if diagnosed early.

## Investigation and Outcomes

On January 19, 2024, a 2-year-old cat began receiving veterinary care in Central Oregon for a neck abscess and vomiting. The cat was initially treated with an oral antibiotic. On January 24, the abscess was excised and drained by a veterinarian.

On January 25, the cat’s owner, a man aged 73 years, cut his right index finger with a kitchen knife and sought treatment at an urgent care facility in Central Oregon. The wound was irrigated and sutured, and the man returned home. That same day, the man had close contact with his cat, which was still under veterinary care. On January 26, the man noticed a new, tender, raised ulcer on his right wrist, and 4 days later he sought care at an emergency department. His signs and symptoms included cellulitis and lymphadenitis extending up to his right axilla, originating from the ulcerated wrist lesion. He was admitted to the hospital and initially treated with empiric intravenous ceftriaxone and metronidazole for bacterial lymphangitis. *Y. pestis *was detected by blood culture evaluated at the hospital microbiology laboratory and was confirmed by polymerase chain reaction (PCR) and bacteriophage-lysis testing at the Washington State Public Health Laboratory on February 6. At the time the isolate was identified, Oregon State Public Health Laboratory was undergoing renovations and Washington State Public Health Laboratory agreed to support and perform pathogen testing by PCR as needed.

Based on culture results, antibiotic therapy was changed to intravenous gentamicin and intravenous levofloxacin, which resulted in improvement in the patient’s cellulitis, lymphangitis, and lymphadenitis. On February 7, he was discharged and prescribed a 9-day course of oral levofloxacin. At his follow-up appointment on February 15, he appeared to have made a full recovery, with only mild residual fatigue.

The owner was not able to give the cat its antibiotics after surgery, and the cat died on January 31. The Washington State Public Health Laboratory reported the man’s positive *Y. pestis* test results to the Oregon State Public Health Laboratory and Oregon’s public health veterinarian, who contacted CDC to request confirmatory testing of the cat. CDC’s Diagnostic and Reference Laboratory in Fort Collins, Colorado, requested tissue sections from the cat’s liver and spleen and subsequently confirmed the presence of *Y. pestis* via PCR and tissue culture.

This project is classified as a public health surveillance activity conducted, supported, requested, ordered, required, or authorized by a public health authority (e.g., Oregon Health Authority). Per federal regulations, this activity does not constitute research involving human subjects and is therefore not subject to institutional review board review.

## Conclusions and Actions

This human case of plague occurred earlier in the calendar year than the other 18 cases reported in Oregon during the previous 90 years. Vectorborne diseases can emerge or reemerge with changes in climate, soils, forest cover, and land use (*1*). Temperate climates of California’s Central Valley and the Pacific Northwest can be conducive to flea emergence year-round, and various factors, such as unseasonal warm temperatures during the winter, can extend the flea life cycle and potentially promote enzootic transmission (*2*). Flea eggs can hatch in temperatures as low as 50°F (10°C) (*3*), which was close to the average temperature in Central Oregon when the cat became ill. The effect of environmental factors, including climate, on plague transmission remains an area of active research (*2**,**4*).

*Y. pestis* can be transmitted to humans through exposure to ill pets, especially cats (*5*). Regular treatment of pets and their surroundings for fleas might reduce the risk for infection with pathogens transmitted by fleas. *Y. pestis* infection was not considered during the cat’s veterinary screening. Had it been, the pet owner could have been counseled about the risks of animal-to-human plague transmission, potentially preventing zoonotic spread. Veterinarians and medical personnel should maintain a high index of suspicion for *Y. pestis* infection in patients with a febrile illness associated with animal exposure in areas where *Y. pestis* is enzootic, regardless of season.
